# A Friend or Foe: Understanding the Physiological Significance, Therapeutic Uses, and Potential Risks of Glycerophosphocholine—A Narrative Review

**DOI:** 10.3390/nu18101526

**Published:** 2026-05-11

**Authors:** Siyi Chen, Takeshi Ohkubo, Noriyuki Yanaka, Rahmawati Aisyah

**Affiliations:** 1 Graduate School of Integrated Sciences for Life, Hiroshima University, Hiroshima 739-8528, Japan; schen6200@gmail.com; 2 Faculty of Human Sciences, Sendai Shirayuri Women’s College, Sendai 981-3107, Japan; t-ohkubo@sendai-shirayuri.ac.jp

**Keywords:** glycerophosphocholine (GPC), trimethylamine *N*-oxide (TMAO), GPC supplementation, GPC absorption

## Abstract

Glycerophosphocholine (GPC) is a well-known choline-containing compound commonly found in nature and has demonstrated potential therapeutic effects in aging-related conditions, such as neurodegenerative disorders, skeletal muscle performance, and eye disorders. Despite being widely used as a supplement, the mechanism by which GPC is absorbed and metabolized in the digestive system remains elusive. Furthermore, growing evidence suggests that high intake of choline-containing compounds, including GPC, is linked with trimethylamine *N*-oxide (TMAO) production, a metabolite associated with atherosclerosis progression. However, there has been an inconsistency that is not commonly discussed, and thus, the adverse effect of TMAO remains debatable. These warrant a better understanding of the physiological significance and metabolism of GPC in the body and how it is linked to TMAO and its potential risk. Through a comprehensive literature search, this narrative review aims to fill these gaps by providing a summary of the physiological significance and supplementation evidence of GPC. Further, the review also highlights the absorption mechanisms and relationship of GPC with intestinal microbiota and its relationship with TMAO production. Lastly, this review addresses and discusses the challenge of GPC supplementation and provides a brief view on future perspectives on GPC as a bioactive compound.

## 1. Introduction

Globally, the human lifespan has sharply increased owing to the advancements in the environment, food, and medicine. As life expectancy gradually increases, the number of older people (>65 years old) continues to grow, and is expected to reach over 1.5 billion people by 2050 [1,2]. Consequently, the prevalence of aging-related diseases is also increasing, posing a challenge for healthcare systems in many countries. In the last three decades, research on aging has progressed impressively, closing the gap between lifespan and healthspan, which refers to the period free from diseases [3]. Currently, drug treatments, such as metformin and rapamycin, and non-drug treatments, such as nutritional interventions and modulation of the gut microbiota, have received much interest [4,5,6]. Despite the efficacy of drug therapy, it presents several risks, such as metabolic dysfunction and vitamin B12 deficiency, raising concerns regarding its widespread use [7,8]. In contrast, bioactive compounds found in natural foods, such as polyphenols, sterols, and polysaccharides, have been shown to attenuate aging-related diseases [9,10], suggesting nutritional supplements as a safer, more promising intervention against aging [3]. Hence, the search for bioactive compounds and functional foods with anti-aging strategies has been a hot spot in aging research.

Glycerophosphocholine (GPC) is a well-studied bioactive compound in aging-related diseases, particularly those related to brain and cognitive disorders. It is a water-soluble, choline-containing glycerophosphodiester (C_8_H_20_NO_6_P) and has a molecular weight of 257.22 g/mol [11]. GPC is naturally generated in the human body from the hydrolysis of phosphatidylcholine (PC), in which the fatty acids of PC are cleaved by phospholipase, resulting in the glycerol backbone and phosphorylcholine group [12]. It is an intermediate in choline-related pathways, which are involved in the production of betaine (methyl donor), PC (membrane phospholipids and component of bile and lipoprotein), and acetylcholine (neurotransmitter) [13,14,15,16]. In foods, GPC is mainly found in animal products such as fish and milk, and oats. It is also regarded as the most used choline supplementation owing to its high choline content (~41% by weight) and its ability to cross the blood–brain barrier [11].

Several animal and human clinical studies have shown that GPC supplementation improves cognitive and behavioral outcomes in neurodegenerative disorders, possibly by acting as an acetylcholine precursor [17,18]. Furthermore, GPC shows potential benefits in improving skeletal muscle performance [19,20], alcohol-induced liver disease [21], ocular health [22], and listening comprehension [23], all of which commonly occur in the elderly, suggesting its potential benefits as a therapeutic agent for aging-related diseases. GPC is commercially available and widely used as a dietary supplement in several countries, including Canada, Italy, Japan, Russia, South Korea, and the United States [24]. Despite its extensive use as a supplement, the mechanism by which GPC is absorbed and metabolized remains obscure [25]. Moreover, recent research suggests that choline-containing compounds, including GPC, are metabolized into trimethylamine (TMA) by gut microbiota, which is subsequently absorbed and converted into trimethylamine *N*-oxide (TMAO) in the liver [26]. This raises concerns as TMAO has been receiving attention for its proatherogenic properties [27,28,29]. Considering the growing number of studies linking TMAO levels to the risk of cardiovascular diseases [26,27,28,30], it is necessary to further understand the physiological significance and metabolism of GPC in the body. Furthermore, there have been inconsistencies in the studies related to TMAO and cardiovascular diseases, which are often overlooked in the discussion regarding its adverse effects. Therefore, this narrative review aims to summarize the current state of knowledge about GPC, from its physiological significance, evidence of supplementation, absorption, and relationship with the gut microbiota and TMAO production, and discuss its potential benefits and risks as nutritional therapy.

## 2. Methods

We constructed this narrative review through a comprehensive literature search of PubMed/MEDLINE for English-language publications up to February 2026. Search strategies used combinations of free-text keywords related to GPC, choline, TMAO, and aging-related conditions. Representative keywords include “Glycerophosphocholine”, “Choline alfoscerate”, “GPC”, “choline”, “TMAO”, “neurodegenerative disease”, “muscle function”, and “cardiovascular diseases”. Due to the narrative structure, we did not employ formal systematic review procedures, such as PRISMA-based selection or quantitative quality scoring. However, we included elements of structured evidence identification and thematic categorization to improve transparency and methodological accuracy. Selection of relevant papers was conducted through a stepwise screening of the titles, abstracts, and full texts. Literature inclusion was guided by thematic relevance and conceptual contribution, which includes physiological roles of GPC and choline, evidence of GPC supplementation (preclinical and clinical), and choline-associated TMAO production. Articles in a language other than English were excluded to avoid misinterpretation due to language barriers. In addition, those with low relevance were also excluded. Eligible articles were carefully reviewed, and key articles were chosen based on the relevancy with the themes mentioned. Additional relevant articles were identified through reference screening of key articles. To address the potential bias due to the narrative nature of the review, we applied a predefined conceptual structure, ensured thorough coverage of major areas, and prioritized high-quality evidence.

## 3. Physiological Significance of GPC: Utilization in the Body

**Figure 1:**
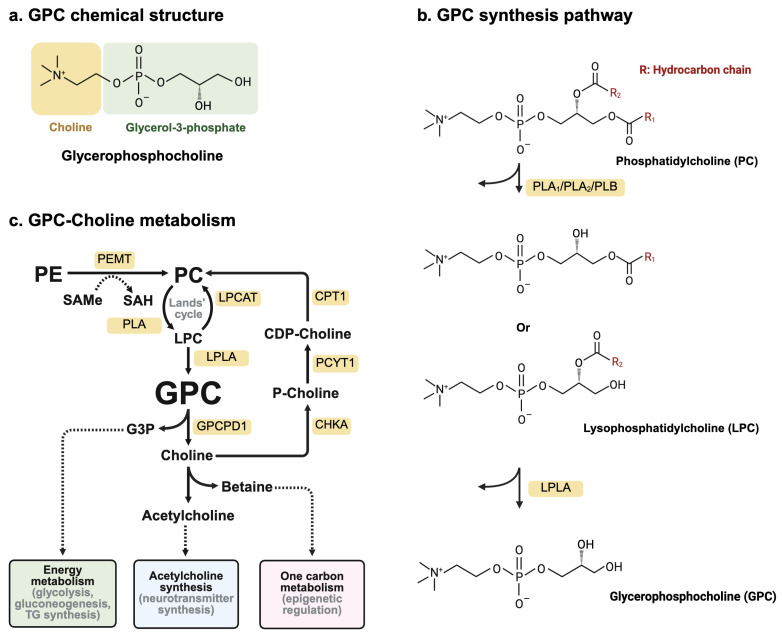
GPC chemical structure and metabolic pathways. (**a**) GPC structure. (**b**) GPC–choline metabolism. (**c**) GPC synthesis pathway. CHKA, choline kinase alpha; CPT1, choline phosphotransferase 1; G3P, glycerol-3-phosphate; GPCPD1, glycerophosphodiesterase 5; LPC, lysophosphocholine; LPCAT, lysophosphatidylcholine acyltransferases; LPLA, lyso-phospholipase-A1; PCYT1, Phosphocholine cytidylyltransferase 1; PC, phosphatidylcholine; PE, phosphatidylethanolamine; PEMT, phosphatidylethanolamine *N*-methyltransferase; PLA, phospholipase; SAH, *S*-adenosylhomocystein; SAMe, *S*-adenosylmethionine.

GPC was first discovered in canine renal medullas treated with antidiuresis by Karl Ulrich in the 1950s [31]. It is a major water-soluble form of choline storage and is recognized as an intermediate in PC metabolism, which is a main component of the eukaryotic phospholipid membrane [13]. In this pathway, PC hydrolysis mainly relies on phospholipase activity, by which PC is degraded into lysophosphocholine (LPC) by phospholipase A_2_ (PLA_2_), and subsequently deacetylated into GPC and free fatty acids by lysophospholipase A_1_ (lyso-PLA_1_), which results in the glycerol backbone and phosphorylcholine group (Figure 1a,b). GPC is further hydrolyzed into free choline and glycerol-3-phosphate by the glycerophosphodiesterase (GDE/Gpcpd) family of enzymes. The choline produced can be shuttled back into the CDP–choline pathway for PC synthesis, oxidized into betaine as a methyl donor in one-carbon metabolism, or converted into acetylcholine for neurotransmitter production (Figure 1c) [13,14,15]. GPC contains glycerol-3-phosphate linked to choline, and the absence of acyl chains makes it a highly polar metabolite unable to integrate into lipid bilayers [12]. Thus, GPC functions as a soluble intermediate in phospholipid hydrolysis and a readily mobilizable reservoir of choline.

As a precursor of choline, GPC engages in various biological processes in the body through its role as an intermediate in the choline metabolic pathway. In the liver, GPC is mainly involved in choline metabolism for betaine synthesis, a methyl donor that links choline to the one-carbon metabolism, and for PC synthesis, which is important for the assembly and/or secretion of lipoprotein and for solubilizing cholesterol in bile [32,33]. PC-containing bile and lipoproteins play essential roles in lipid homeostasis, by which the secreted PC forms mixed micelles with bile acids and cholesterol, protecting the biliary epithelium and facilitating lipid digestion [34,35]. Hepatic PC is particularly important for lipoprotein metabolism, as it affects the activity and expression of elements that modulate hepatic lipoprotein generation, including apo B degradation and expression of ABCA1 and the scavenger receptor B1 [36]. Furthermore, hepatic PC turnover through bile and very-low-density lipoprotein (VLDL) accounts for more than 50% of the hepatic PC pool per day, emphasizing the significance of choline supply for liver function and lipid metabolism [16]. Choline deficiency leads to the development of fatty liver, possibly due to disrupted production of VLDL, the primary reservoir that secretes triglycerides in the liver [36]. Thus, GPC may serve as an abundant source of choline to prevent fatty liver and maintain hepatic choline metabolism, emphasizing its significance in maintaining liver function.

In the kidney, GPC mainly acts as an osmolyte. Renal medullary cells contain the highest concentrations of organic osmolytes, owing to their roles in concentrating the urine, which exposes them to extremely high levels of NaCl and urea [37]. GPC is one of the organic osmolytes in renal medullary cells, in addition to betaine, inositol, and taurine [38]. It was confirmed as an osmotically active organic compound by ^14^N nuclear magnetic resonance (NMR) in rabbit and rat kidneys, thirty years after it was initially discovered [39]. GPC is unique among the renal medullary organic osmolytes as it accumulates in response to high NaCl and urea, as well as hypertonicity [40,41]. This was confirmed through experiments using Madin–Darby canine kidney (MDCK) cell cultures, which showed that GPC accumulated following increased osmolality, either from NaCl or urea [42], establishing the physiological importance of GPC as an osmolyte in maintaining cellular function and volume stability in the kidney.

GPC is also involved in neurotransmission through its role as a choline precursor. Choline produced from GPC hydrolysis by GDE/Gpcpd will be used to synthesize the neurotransmitter acetylcholine. This reaction is catalyzed by choline acyltransferase in the cytosol of presynaptic cholinergic neurons. Acetylcholine in both the central and peripheral nervous systems is wrapped into vesicles and released into the synaptic cleft to bind its receptors on presynaptic and postsynaptic neurons. Increased acetylcholine levels have been shown to improve cholinergic transmission, which is disrupted in neurocognitive disorders, suggesting the importance of GPC as a choline source in neurotransmission function [43].

GPC is also possibly involved in sphingomyelin metabolism and sphingomyelin-associated apoptosis through its role as a choline source. Sphingomyelin is a sphingolipid mainly found in the myelin sheath surrounding nerve cell axons, which possibly plays a role in signal transduction and as an insulator of nerve fibers [44,45]. It is synthesized through the enzymatic transfer of phosphocholine from PC to a ceramide by sphingomyelin synthase [46,47]. Choline deficiency leads to decreased PC supply for this process and results in elevated ceramide levels accompanied by increased apoptosis. Given the proapoptotic properties of ceramides, maintaining adequate choline levels may be necessary for apoptotic regulation via sphingomyelin synthesis [48,49]. As a choline precursor and an intermediate in PC–choline metabolism, this suggests that GPC may contribute as an important choline source for apoptotic regulation through modulation of sphingomyelin synthesis.

In addition to its role in various tissues, GPC has emerged as an attractive target in diseases where abnormal choline metabolism is observed, owing to its role as an intermediate in choline metabolism. For example, Alzheimer’s disease (AD) has been associated with elevated PLA_2_ activity and PC breakdown. However, as it is difficult to confirm this association in vivo, GPC, a water-soluble metabolite involved in PC degradation that can be detected in cerebrospinal fluid (CSF), was chosen as an indicator of PC degradation. In this study, GPC accumulation was observed in CSF of the AD patients, confirming the increased PC breakdown as a characteristic of this disease [50]. A recent study on Huntington’s disease, which has been associated with dysregulated choline metabolism, reported an increase in GPC level in the mouse model of the disease [51]. This suggests a potential role of GPC as a biomarker for diseases related to altered PC–choline metabolism.

GPC has also received attention in cancer research. Aberrant choline metabolism has emerged as one of the hallmarks of oncogenesis [52]. Cancer is one of the leading causes of death; therefore, research focusing on disease detection and management has remained an important topic for decades. Magnetic resonance spectroscopy, a non-invasive imaging technique used to quantify metabolites in the tissues, is capable of detecting phosphocholine, GPC, and free choline or their overlapping signals, commonly referred to as total choline in tissues [53,54]. Studies using this method, particularly in breast cancer models, have shown that a decrease in GPC and an increase in phosphocholine levels are common characteristics of malignancy, and the levels were reversed following treatment with chemotherapeutic agents [15,55,56]. Furthermore, changes in GPC and phosphocholine levels following treatment can be evaluated non-invasively as early as 24 h after the first treatment [57,58,59], suggesting that the GPC/phosphocholine ratio may serve as a promising biomarker for both disease progression and treatment effectiveness in breast cancer. However, in other cancers, different profiles have been observed, indicating that the changes in choline metabolite profiles may depend on the type of cancer [60,61,62]. It remains unclear why choline metabolites are altered in cancer cells; however, it can be speculated that the uncontrolled cell growth may affect the metabolism of membrane phospholipids, leading to abnormal choline utilization within the cell [52]. Although the profile trends of choline metabolite levels may be dependent on cancer type, these findings suggest that GPC is a promising, non-invasive imaging biomarker for cancer diagnosis, prognosis, and treatment monitoring. Overall, GPC may offer potential benefits to various biological processes through its role as a precursor of choline and an intermediate in choline metabolism.

## 4. GPC Supplementation: Accumulating Evidence

Owing to the significant roles of choline in various physiological processes, maintaining sufficient choline intake is essential for homeostasis. Dietary choline deficiency has been shown to cause several health consequences, such as fatty liver and increased risk of having a baby with neural tube defects (NTD) [63,64,65,66]. It has also been observed in pathological conditions, including cystic fibrosis patients with exocrine pancreatic insufficiency and patients undergoing parenteral nutrition [67,68]. Despite its physiological significance, epidemiological studies have shown that choline intake below the adequate intake (AI) is prevalent in developed countries [69,70,71], suggesting that the nutritional importance of choline is under-recognized. GPC, also known as choline alfoscerate or α-GPC, is a water-soluble cholinergic compound widely used as a dietary supplement. It is also one of the major choline compounds found in breast milk (40% of total choline in breast milk), making GPC a main natural choline source in breast-fed infants and suckling animals [72,73]. The high concentration of GPC in breast milk likely reflects the infant’s high demand for absorbable choline, because infant blood choline levels are positively correlated with GPC in breast milk [74]. Owing to its abundance in nature and its high choline content (approximately 41% by weight) [11], GPC is one of the most potent choline sources to ensure sufficient choline intake. Extensive preclinical and clinical studies have demonstrated the pharmacological benefits of GPC under various conditions, which are summarized in Table 1 and Table 2, respectively.

Evidence on the neuroprotective effects of GPC is particularly abundant, with most studies showing improvements in aging-related cognitive impairments. In vitro studies using SH-SY5Y cells treated with amyloid β-protein (Aβ), the main component of amyloid plaque found in the brain of AD, reveal that the addition of GPC increases the cell survival rate, indicating that GPC may protect cells from Aβ-induced cytotoxicity [85,87]. This neuroprotective effect of GPC was further confirmed in various in vivo models [75,76,77] and clinical studies [93,94,105,106], in which GPC supplementation alleviated AD-associated cognitive impairments. The favorable effect of GPC on cognitive function also extends to impairments caused by other conditions, such as vascular dementia [91,107,108,109], hypoxia/ischemia [99,110], stress [89], and epilepsy [83]. Cognitive deficits have been associated with a decline in cholinergic neurotransmission. As a precursor of acetylcholine, GPC may exert its neuroprotective properties by enhancing cholinergic neurotransmission and thus ameliorating cognitive declines.

In addition to its neuroprotective effects, clinical studies have shown that GPC supplementation improves strength and performance and promotes recovery [19,20,97,100]. Phospholipid composition is associated with muscle strength and performance, and our recent study showed that accumulated GPC in skeletal muscle-specific *Gpcpd1*-KO mice leads to altered PC composition and muscle force production [111]. In addition, acetylcholine is involved in neuromuscular signaling, and low choline levels have been associated with strenuous exercise [112,113]. Given that GPC is involved in PC–choline metabolism and is an abundant source of choline, it potentially affects muscle performance through the modulation of phospholipid composition and acetylcholine availability. Although the molecular mechanism remains to be investigated, GPC offers a promising alternative for the treatment of aging-related declines in muscle function.

In the rat liver, GPC supplementation ameliorated alcohol-induced mitochondrial damage. Alcohol exposure leads to a decrease in the oxidative phosphorylation function of liver mitochondria, and this condition was alleviated with GPC supplementation (oral feeding with 0.8% GPC-enriched diet, 8 days) [21]. Oxidative stress also plays an important role in alcohol-induced liver injury, and in another study, GPC supplementation (intragastric GPC 50 mg/kg, 8 days) attenuated sodium azide liver damage, possibly through its role in suppressing oxidative stress [80]. Additionally, as GPC contains abundant choline compounds, it may serve as an important choline precursor in the liver to maintain liver function. Choline supplementation ameliorates the development of alcoholic liver diseases in mice, possibly through its protective effect in intestinal barrier dysfunction [114]. In a human study, choline supplementation in the form of PC (1200 mg PC, twice/day for 12 weeks) shows favorable effects on hepatic steatosis, oxidative stress, inflammatory markers, liver enzyme levels, and lipid profile in non-alcoholic fatty liver disease (NAFLD) patients [115]. Although direct evidence between liver function and GPC supplementation is relatively scarce compared to other forms of choline supplementation, given its high choline content, GPC can be a promising form of choline supplementation for liver diseases. Future preclinical and clinical studies using GPC are warranted, and the exact mechanisms remain to be investigated.

**Figure 2:**
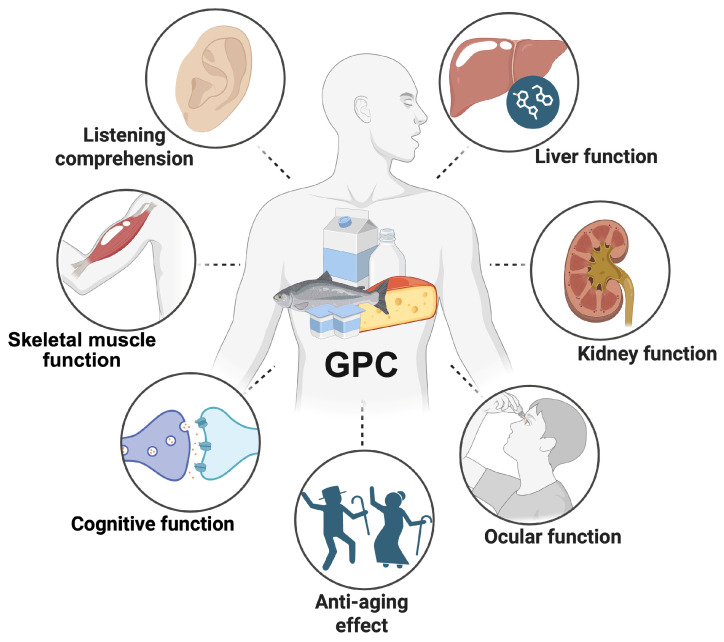
Potential health benefits of GPC. GPC, L-*α*-glycerylphosphorylcholine.

GPC also leads to improved listening comprehension in older hearing aid users [23] and accelerated corneal innervation post-cataract surgery [22], both of which are often observed in aging populations. Furthermore, a preclinical study in *C. elegans* revealed that GPC enhanced lifespan, improved fitness and stress resistance, and decreased intracellular ROS levels, with no adverse effects on fertility and body length [88]. In old C57BL/6J mice, GPC increases the expression of genes associated with long-term potentiation, which is related to cognitive function, and β-oxidation, which is related to lipid metabolism, but has no effect on taste sensitivity [86]. In the senescence-accelerated mouse prone 8 (SAMP8) mice, an animal model of accelerated aging, GPC decreased transthyretin levels in the brain, which plays a role in neuroinflammation, and improved joint degeneration [84]. These emphasize the compelling features of GPC as a nutritional supplement for aging-related health issues (Figure 2).

## 5. GPC Absorption in Mammals: Role of Gut Microbiota and Gpcpd1

Despite growing interest and evidence in the physiological benefits of GPC supplementation, the mechanisms governing its intestinal absorption and metabolism remain poorly understood. In general, choline absorption occurs mainly in the small intestines and exists in both water-soluble and lipid-soluble forms, each of which follows a distinct metabolic pathway [116]. Water-soluble compounds are transported across the intestinal epithelium via mediated transport. In contrast, lipid-soluble forms, such as PC, are either incorporated into chylomicrons and transported through the lymphatic system or hydrolyzed by pancreatic lipases, generating GPC as an absorbable intermediate [117,118]. Because choline is a quaternary ammonium compound with a permanent positive charge, it cannot passively diffuse across lipid bilayers and, therefore, requires specific transport systems [119,120,121]. GPC, as a water-soluble choline-containing metabolite, has been presumed to undergo transporter-mediated uptake. However, no specific GPC transporters have been conclusively identified. Even so, we previously reported that GPC supplementation at 500 mg/kg through oral gavage increased plasma GPC and choline levels 30 min after administration, which subsequently subsided an hour post-administration [25]. Another study using Apoe^−/−^ mice also reported increases in plasma d9-GPC an hour (female) and 2 h (male) post d9-GPC administration (150 mM, via oral gavage) [122]. These suggest that GPC is rapidly absorbed and metabolized in the body despite no specific transporter being identified yet. However, in a human study, GPC oral intake increases plasma choline level, but it remains unclear if plasma GPC level is affected [123], emphasizing the necessity to further investigate how GPC is absorbed and metabolized in the body.

**Figure 3:**
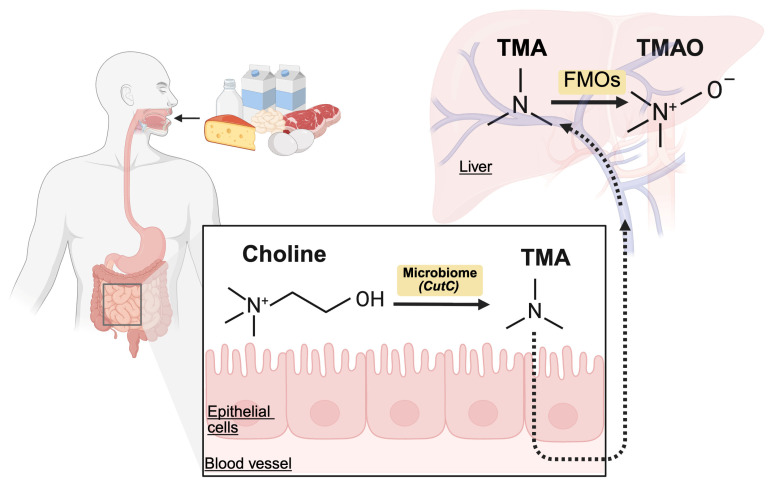
TMAO production from choline-containing compounds. FMO, flavin-containing monooxygenase; TMA, trymethylamine; TMAO, trymethylamine-*N*-oxide.

Parallel to host uptake, the intestinal microbiota competes for luminal choline. Accumulating evidence suggests that choline-containing compounds, including free choline, PC, and GPC, can be metabolized by the gut bacteria into TMA, a volatile tertiary amine that readily crosses epithelial membranes [124,125,126,127]. Once absorbed into portal circulation, it is oxidized in the liver by flavin-containing monooxygenases (primarily FMO3 and, to a lesser extent, FMO1) to form TMAO (Figure 3) [128,129,130]. Approximately 95% of TMA is oxidized into TMAO, cleared by the kidneys, and excreted in the urine at a 3:95 TMA:TMAO ratio. TMA-producing gut bacteria mainly reside in the distal part of the small intestine, cecum, and colon [131]. The incomplete hydrolysis and absorption of GPC in the proximal intestine may serve as a substrate for microbial TMA generation, thereby influencing systemic TMAO levels. Thus, TMA and TMAO production are believed to depend on both substrate availability and microbial activity.

The important roles of gut microbiota in TMAO formation from choline-containing compounds were demonstrated by Tang et al. [132], who showed that antibiotic treatment in healthy humans suppressed plasma TMAO levels following a PC challenge (two large, hard-boiled eggs providing approximately 500 mg choline). Mechanistically, studies using *Desulfovibrio desulfuricans* uncovered the TMA synthesis pathway, which involves a radical C–N bond cleavage of choline to produce TMA and acetaldehyde. This experiment also revealed the responsible gene cluster, including *cutC*, which encodes a glycyl radical enzyme with choline TMA-lyase activity, and *cutD*, which encodes a glycyl radical-activating protein responsible for the microcompartment assembly, which may sequester the acetaldehyde byproduct [132,133]. Furthermore, several studies have identified *cutC*/*cutD*-harboring microbiota in human feces, emphasizing the contribution of the gut microbiota to systemic TMA/TMAO production. Both *cutC*/*cutD* genes were distributed across various taxa belonging to Actinobacteria, Firmicutes, and Proteobacteria [124,126]. Using ultra-high-pressure liquid chromatography coupled with tandem mass spectrometry (uHPLC-MS/MS), Romano et al. screened 79 sequenced human intestinal isolates and identified eight species representing two different phyla (Firmicutes and Proteobacteria) and six genera capable of significant choline consumption and TMA production, including: *Anaerococcus hydrogenalis*, *Clostridium asparagiforme*, *Clostridium hathewayi*, *Clostridium sporogenes*, *Escherichia fergusonii*, *Proteus penneri*, *Providencia rettgeri*, and *Edwardsiella tarda* [134]. Notably, even a relatively small amount (~0.15%) of TMA-producing microbiota within the total microbial community appeared to be sufficient to generate a substantial amount of TMA from choline. For example, studies using human fecal samples have shown that potential TMA producers are particularly associated with *Clostridium* XIVa strains and *Eubacterium* sp. strain AB3007 [133]. The abundance of *cutC*-containing bacteria has also been correlated with urinary TMAO level [135], further supporting the essential role of the gut microbiota in TMA/TMAO production from choline-containing compounds, including GPC, in the human gut.

**Figure 4:**
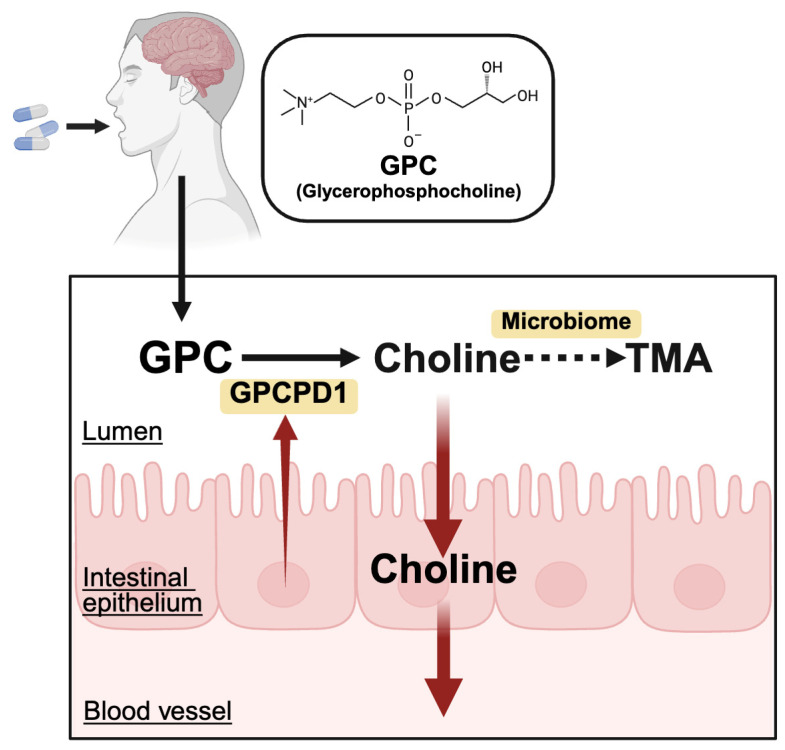
Role of Gpcpd1 in TMAO production.

Although microbial metabolism plays a major role in TMA formation, recent findings suggest that the host epithelium also contributes to GPC processing. In our recent study, we identified an intrinsic mechanism that enhances choline acquisition independently of microbial metabolism [25]. Using Caco-2 cell monolayer experiments, we observed that exogenously added GPC was hydrolyzed to choline in the apical medium, and only partial GPC was transferred through the intracellular spaces between enterocytes. These findings indicated that GPC underwent luminal hydrolysis prior to absorption. We further identified the cytosolic enzyme Gpcpd1 as a key mediator of this process. GPCPD1 can translocate to the extracellular space and facilitate luminal GPC hydrolysis. In intestinal epithelial-specific *Gpcpd1*-knockout mice, *Gpcpd1* deletion affected intestinal GPC metabolism and partially attenuated the increase in circulating TMAO levels induced by GPC administration [25]. These findings suggest that TMAO production from GPC is not solely dependent on the gut microbiota, but it is also influenced by host GPCPD1 activity (Figure 4).

The interplay between the gut microbiota and intestinal GPCPD1 activity likely indicates a balance between host choline acquisition and microbial TMA production. Further investigations are necessary to clarify how this host–microbe competition regulates GPC bioavailability and how it ultimately impacts the physiological and pathological consequences of GPC supplementation. In addition, the use of GPC is not limited to oral supplementation but also parenteral, which bypasses intestinal metabolism and is therefore expected to deliver GPC directly into circulation. However, the pharmacokinetics of GPC and how GPC contributes to systemic choline availability, whether it is circulated as intact GPC or mainly converted into choline, remain unclear. Thus, further study is also warranted.

## 6. Challenge in GPC Supplementation: TMAO Production

Numerous preclinical and clinical studies have reported that GPC is well-tolerated and has little to no adverse effects, highlighting its safety as a food supplement. Brownawell et al. [136] conducted a safety study for GPC supplementation in various animal models and reported that the oral LD50 in rodents was ≥10,000 mg/kg, with preceding convulsions before death. In dogs, treatment with 3000 mg/kg GPC resulted in reduced activity. Subchronic and chronic administration of 1000 mg/kg/day in rats and 300 mg/kg/day in beagles led to reduced activity, minor decreases in food intake and weight gain, and slight decreases in liver weight, accompanied by significant decreases in plasma alkaline phosphatase, bilirubin, and triglycerides. No histopathological correlations were observed, and both in vivo and in vitro assays suggested no mutagenicity of GPC. A multicenter clinical trial on 120 patients with vascular dementia shows that 1000 mg/day of GPC for 90 days resulted in no adverse effects [91]. An open multicenter trial of 2044 individuals with recent transient ischemic attacks or stroke used oral GPC at 1000 mg/day for 28 days, followed by 400 mg t.i.d. in the following 5 months. Adverse effects were reported in 44 patients (2.14%), with the most common complaints being heartburn (0.7%), nausea/vomiting (0.5%), insomnia/excitation (0.4%), and headache (0.2%) [18]. Another study of 65 subjects with probable senile dementia of the Alzheimer’s type using oral GPC at 1200 mg/day (800 mg in the morning and 400 mg in the afternoon) for 6 months showed that only one person reported insomnia, gastralgia, and restlessness [93]. These reports demonstrate the favorable safety and excellent tolerability of GPC supplementation.

Despite a large number of studies reporting on the beneficial effects and safety of GPC, there has been an emerging concern regarding the potential side effects associated with the risk of cardiovascular disease (CVD). As mentioned in the previous section, GPC, along with other choline-containing compounds, can be metabolized by the gut microbiota to produce TMA, which is subsequently oxidized in the liver to produce TMAO. An increasing number of reports have shown that TMAO levels may be correlated with CVD risk. In a clinical cohort study employing a metabolomic approach, Wang et al. [27] revealed that three metabolites of the dietary lipid PC (choline, TMAO, and betaine) were strongly associated with the risk of CVD. In line with this result, choline or TMAO-enriched diets aggravated atherosclerosis development in atherosclerosis-prone mice, and this effect was dependent on the gut microbiota [137]. A prospective clinical study demonstrated that PC consumption was associated with gut microbiota-dependent formation of TMAO, and that higher plasma TMAO levels were associated with a higher risk of adverse cardiovascular events [132]. A large cohort study involving 120,412 individuals also reported that a higher PC intake was associated with increased risks of all causes and CVD mortality, and this association was stronger in participants with diabetes [28]. Another large cohort study by Lee et al. [29], which included more than 12 million individuals, suggested that GPC supplementation was correlated with an elevated 10-year incident stroke risk in a dose-dependent manner. Following this striking report, an animal study employing atherosclerosis-prone Apoe^−/−^ mice fed with a diet supplemented with 1% GPC for 16 weeks showed that GPC promotes atherosclerosis, possibly through increasing TMAO biosynthesis, shifting gut microbiota composition, and thereby reducing SCFA production and increasing pro-inflammatory factors, and activating MAPK and NF-kB signaling in artery endothelial cells [122]. In another atherosclerosis-prone mouse model, Ldlr^−/−^, chronic choline supplementation elevated plasma TMAO levels and upregulated gene expressions related to inflammation in vascular cells [138]. These studies suggest that prolonged consumption of GPC may lead to elevated CVD risk through TMAO production. In addition to CVD risk, emerging evidence also showed that TMAO levels were inversely related to cognitive performance in older adults, and higher TMAO in old mice was correlated with higher brain pro-inflammatory cytokines and markers of astrocyte activation [139]. In another study, TMAO exposure significantly impaired cognitive function in young mice [140]. These further raise questions about the safety and effectiveness of GPC as a food supplement and pharmacological agent.

TMAO was initially only considered a waste product of choline metabolism in humans. However, it is now recognized as a biologically active compound, which serves as an osmolyte and a molecular chaperone that maintains the folded state of proteins and stabilizes them against denaturants [131]. Although there is compelling evidence that increased TMAO levels and diets rich in its precursors (choline-containing compounds) are associated with a higher CVD risk, these inconsistencies are just as strong. Two different prospective studies with 14,430 middle-aged individuals [141] and 16,165 women aged 45–70 years old [142] showed that dietary choline and betaine, the precursors of TMAO, were not associated with CVD risk. A systematic review has elaborated that egg consumption, which is rich in TMAO precursors, is not correlated with the risk of CVD and cardiac mortality [143]. Another prospective study in Japan with more than 25,000 participants also reported no correlation between choline or betaine intake and CVD mortality risk [144]. A cross-sectional study also showed that plasma TMAO levels did not differ significantly between patients with coronary artery disease and controls [145]. In several countries, including Canada, Italy, Japan, Russia, South Korea, and the United States, GPC is commercially available as a pharmaceutical product or dietary supplement and is often prescribed to elderly individuals to prevent cognitive decline. Thus, it is possible that the participants consuming GPC in studies that reported a positive correlation between GPC and CVD risk might have already had underlying CVD or subclinical atherosclerotic changes by the time the studies were conducted, possibly influencing the results. The Coronary Artery Risk Development in Young Adults (CARDIA) study evaluated the role of TMAO in early atherosclerosis development and found no association between TMAO levels and atherosclerosis development. With relatively younger and healthier subjects (age 33–45 years old, *n* = 817), confounding factors such as age-related diseases or subclinical CVD were minimal in this study [146].

In animal studies, although chronic choline supplementation leads to elevated TMAO levels and atherosclerosis progression in Apoe^−/−^ and Ldlr^−/−^ mice, studies employing APOE*3-Leiden.CETP and CETP-expressing Apoe^−/−^ mice showed that neither choline nor TMAO affect atherosclerosis development [147,148]. Notably, wild-type mice do not have cholesteryl-ester transfer protein (CETP), which transfers cholesterol ester from high-density lipoprotein (HDL) to low-density lipoprotein (LDL) [149]. APOE*3-Leiden.CETP mice possess human CETP, and in these mice, choline mediates an increase in CETP-mediated transfer of neutral lipid between lipoproteins and clearance of triglyceride-rich lipoprotein through APOE-LDLR, both of which are functional compared to those of Apoe^−/−^ mice. This suggests that the proatherogenic properties of TMAO may not be significant because it depends on the mouse model. Furthermore, although long-term GPC supplementation affected the progression of atherosclerosis in Apoe^−/−^ mice, it is unclear if similar results can be reproduced in normal mice. In addition, Zeisel and Warrier, in their elegant review on TMAO, argued that the dietary choline and/or TMAO employed in some studies were too high and may not be physiologically relevant to be translated into human studies, especially since high choline levels can be toxic for humans [131].

Taken together, although there is growing evidence that GPC supplementation may be related to increased CVD risk due to TMAO production by the gut microbiota, in both human and animal studies, the conflicting results are equally compelling. Given the widespread use of GPC as a dietary supplement, further investigation is warranted to clarify the relationship between GPC consumption, TMAO production, and CVD risk. Moreover, a study in rats showed that only approximately 6–16% of ingested choline reaches the cecum and colon, where TMA-producing gut bacteria mainly reside, and large increases in TMA and TMAO levels were only observed under a high dose of choline (15 mmol/kg) [26]. Other studies also show that approximately 14% of total choline is converted to TMAO after consumption of egg yolk [150], while adequate PC intake has little to no effect on plasma TMAO levels in healthy individuals [151,152]. GPC oral administration, on the other hand, can raise TMAO levels but not to the extent of PC ingestion [27]. Also, in male Sprague–Dawley rats, dietary PC, unlike GPC or choline (choline chloride), does not increase plasma TMAO levels [153]. These indicate that the amount and forms of dietary choline may be key determinants for its conversion to TMAO. Although in some cases, such as in individuals with small intestine bacterial overgrowth (SIBO), choline degradation to TMA was observed in the small intestine, accompanied by an inverse relationship between plasma choline and TMAO [154,155]. This suggests the necessity to determine the optimal dose where GPC can be effectively absorbed and utilized, and to clarify the contribution of microbial intestinal colonization to choline-derived TMAO production. In addition, TMAO production from GPC may not exclusively depend on the gut microbiota but also on the host GPCPD1 enzyme, presenting an attractive area for drugs and/or food combinations to optimize the GPC effect while minimizing TMAO production.

## 7. Conclusions and Future Perspectives

The current study presents the progress made in understanding GPC, a unique choline metabolite widely found in nature, from its significance in diverse biological processes, metabolism, and interactions with the gut microbiota, and its potential as a dietary supplement. GPC exerts its physiological effects through its role as a choline precursor, which is involved in the biosynthesis of cell membranes, synthesis of PC-containing secretions such as bile and lipoproteins, production of neurotransmitters, and DNA methylation, and through its properties as an organic osmolyte. GPC supplementation has been widely recognized, with most evidence pointing towards its benefits in alleviating neurodegenerative disorders. GPC also shows potential for skeletal muscle health and age-related sensory loss, and preclinical studies have suggested that GPC may prolong lifespan. Given the global demographic shift toward an aging population, GPC is an attractive bioactive compound for aging-related diseases. GPC metabolism is believed to be strongly associated with the intestinal microbiota, where excess GPC is converted to TMA and subsequently oxidized into TMAO by the liver; however, a recent study suggested that host intestinal enzymes may partially play a role in its absorption and metabolism. Despite extensive studies demonstrating the benefits and safety of GPC, concerns regarding its side effects have recently arisen, owing to its potential relationship with increased CVD risk, possibly through TMAO production. Further investigations are warranted to clarify the safety and potential side effects of GPC supplementation, considering the remarkable properties of GPC as a nutritional supplement, especially for aging-related health issues. In addition, the recent updates on GPC absorption and its relationship to the gut microbiota may open a new avenue for developing functional food–drug combinations for human health. As such, GPC will continue to receive much attention as a bioactive compound and provide an attractive research opportunity for the development of therapeutic strategies against various diseases.

## Figures and Tables

**Table 1:** Preclinical evidence of GPC supplementation.

Reference	Animal Model	Dose	Duration	Results
[75]	Adult male SD rats, scopolamine-induced amnesia	i.g. GPC 300, 600, 1000, 2000 mg/kg	2 h pre scopolamine induction	GPC reverses the amnesia caused by scopolamine in passive avoidance test.
[17]	4 and 24 mo male Wistar, scopolamine-induced amnesia	i.p. GPC 25, 50, 100, and 200 mg/kg	2 h pre-test and 21 d	GPC improves behavioral and biochemical parameters in young and aged rats.
[76]	24 mo male SD rats, pharmacologically induced amnesia	i.p. GPC 100 mg/kg/d	20 d	GPC improves learning and memory capacity of animals in all experimental groups.
[77]	Adult male SD rats, scopolamine-induced amnesia	Oral GPC 100–600 mg/kg/day	3 h pre-test	GPC prevents scopolamine-induced amnesia in a time- and dose-dependent manner.
[78]	23 mo SD rats	i.p. GPC 200 mg/kg/d	30 d	GPC restores the number of muscarinic receptors to levels of young animals and partially attenuates membrane microviscosity, hence increasing membrane fluidity.
[79]	Adult mongrel rats induced with ischemia	i.p. GPC 45 mg/kg/d	3 d after ischemia	GPC increases the tolerance of neurons to ischemic damage and slows the execution of the cell death program. GPC also improves cerebral ischemia-induced neurobehavioral deficits.
[80]	SD rats, sodium azide-induced hepatic hypoxia	i.g. GPC 50 mg/kg	8 d	GPC restores sodium azide-induced ATP decrease and liver damage.
[81]	Adult male SD rats exposed to hippocampal radiation	Oral GPC 50 mg/kg/d	1 wk pre-irradiation, 3x/wk (Mon, Wed, and Fri), continued for 4 consecutive mo	GPC improves radiation-induced memory impairment and tissue damage.
[82]	Adult male SD rats exposed to hippocampal radiation	i.v. GPC 50 mg/kg	5 min pre-radiation	GPC supplementation alleviates irradiation-induced peripheral pro-inflammatory activation and ATP depletion.
[83]	8 wk male SD rats, pilocarpine injection to induce status epilepticus	i.m. GPC 250 mg/kg/d	Immediately after seizure onset for 1 or 3 wk and from 3 wk after the seizure onset for 3 wk	Administration of GPC starting at 3 wk after seizure improved cognitive function through reduced neuronal death and BBB disruption, and increased neurogenesis.
[84]	3 wk SAMP8 mice	0.07 mg/mL GPC in drinking water	41 wk	GPC reduces the deposition of transthyretin involved in neuroinflammation and improves joint degeneration.
[85]	SH-SY5Y neuronal cell line	0.1 mM, 1 mM, 10 mM, 50 mM GPC	(1) Cells were incubated in 10 μM Aβ1–42 for 1 h, followed by addition of several concentrations of GPC and kept for 24 h (2) Cells were incubated with several concentrations of GPC for 24 h, followed by addition of 10 μM Aβ1–42, and kept for another 24 h	GPC alleviates the adverse effect of β-amyloid in both in vitro models through the activation of the neurotrophin survival pathway, reduces apoptotic cell death and preserves the neuronal morphology.
[86]	105–118 wk male C57BL/6J	0.0136% GPC-containing solid diet	From the age of 60 to 105 wk	GPC supplementation increases the expression of genes related to long-term potentiation in the hippocampus, which is necessary for the development of memory and learning capability.
[87]	SH-SY5Y neuronal cell line, differentiated for 1 wk with 10 μm of all-trans-retinoic acid, treated for 72 h with 10 μM Aβ25-35	100 nM GPC	1 h pre-treatment	GPC alleviates Aβ25-35-mediated neurotoxicity and attenuates the Aβ-induced phosphorylation of the Tau protein, possibly through the NGF/TrkA system and sustaining the expression level of synaptic vesicle proteins, such as synaptophysin.
[88]	*C. elegans*	10 mM and 50 nM GPC	7 d and 15 d adulthood	GPC promotes lifespan and improves exercise capacity during aging with no adverse effect on nematodes’ reproductive abilities and body length. GPC also enhances the stress resistance of *C. elegans* and inhibits the ROS accumulation in worms.
[89]	8 wk male Wistar rats induced with noise and restraint stress (3 h, 7 d)	Oral GPC 400 mg/kg/d	12 d from the start of stress induction	GPC shows protective effects on stress-induced cognitive dysfunction by promoting neuronal differentiation.

Abbreviations: GPC, L-*α*-glycerylphosphorylcholine; i.g., intragastric(ly); i.m., intramuscular(ly); i.p., intraperitoneal(ly); i.v., intravenous(ly); ROS, reactive oxygen species; SD, Sprague–Dawley.

**Table 2:** Clinical evidence of GPC supplementation.

**Reference**	**Study Design**	**Subject**	**Dose**	**Duration**	**Effects**
[90]	RCT	32 individuals, scopolamine-induced amnesia	Oral GPC 1200 mg/d	10 d	GPC shows rapid onset of memory improvement.
[91]	Open multicenter uncontrolled trial	120 individuals, VaD, (50–80 yo)	i.m. GPC or CDP 1 g/d	90 d	Both result in a definite symptomatic improvement and very good tolerability. GPC produces higher efficacy compared to CDP.
[92]	RCT	112 individuals, multi-infarct dementia (50–80 yo)	i.m. GPC or CDP 1 g/d	90 d	GPC enhances cognitive functions, behavior, and personality at the end of treatment.
[93]	RCT	126 individuals, AD	Oral GPC 1200 mg/day or acetyl-l-carnitine 1300 mg/d	6 mo	Significant improvements in most neuropsychological parameters in GPC recipients. Acetyl-l-carnitine treatment also shows improvement to a lesser extent.
[18]	RCT	2044 individuals, cerebral ischemic attacks within 10 d (45–85 yo)	Parenteral GPC 1000 mg/d (first 28 d) followed by oral GPC 400 mg t.i.d.	6 mo	GPC effectively improves cognitive function in patients with acute cerebrovascular attacks (stroke and/or TIA) with low incidence of adverse effects.
[94]	RCT	261 individuals, AD	Oral GPC 1200 mg/d	180 d	Oral GPC significantly improved cognition and global function in the patients.
[19]	RCT	19 physically active subjects	Oral supplement (1.5 g) containing GPC, choline bitartrate, phosphatidylserine, vitamins B3, B6, and B12, folic acid, L-tyrosine, anhydrous caffeine, acetyl-L-carnitine, and naringin	4 wk	The supplement maintains reaction time and subjective feelings of focus and alertness to both visual and auditory stimuli following exhaustive exercise.
[95]	Open 10-day pilot study	60 individuals, PD (±68 yo)	i.v. GPC 1000 mg/d	10 d	GPC moderately improves the state of cognitive function more often than piracetam. Supplementation was well-tolerated.
[20]	RCT	26 healthy adults	Oral GPC 600 mg/d	6 d	GPC improves isometric mid-thigh pull peak force and lower body force.
[96]	RCT	Children, FASD (5–10 yo)	Oral liquid GPC 5.25 mL (~1240 mg)	6 wk	No significant improvement was observed in memory, attention, executive function, or hyperactivity before and after treatment.
[97]	RCT	48 healthy college-aged males	Oral GPC 500 mg/d, 250 mg/d	7 d	GPC has ergogenic effects at a dose of >250 mg or higher.
[98]	Cohort study	34 individuals, MCI (50–85 yo)	Oral GPC 800 mg/d	3 mo	Tendency of decreased P300 latencies, indicator of cognitive function, following GPC supplementation.
[99]	Cohort study	44 post-stroke patients with depression (45–75 yo)	Oral GPC 600 mg/d	6 mo	GPC slightly improves post-stroke depression symptoms, but not to the extent of SSRI.
[22]	RCT	40 patients undergoing cataract surgery	GPC-containing eye drops (dose undisclosed)	1 mo	GPC-containing eye drops promoted and stabilized the reepithelialization process and accelerated the repair of the corneal innervation.
[23]	Case–control	34 hearing aids users aged 65–85 yo	Oral GPC 800 mg/d	11 mo	GPC effectively enriches listening comprehension in older hearing aid users.
[100]	Crossover RCT	12 overweight or obese women	Oral GPC 1000 mg	1 h before test	GPC consumption recovers HRV and blood pressure faster following strenuous exercise in overweight and obese women.
[101]	RCT	100 individuals, MCI (55–85 yo)	Oral GPC 600 mg/d	12 wk	GPC enhances cognitive outcomes in MCI, and no adverse effects were observed
[102]	RCT	20 healthy adult men	Oral GPC 630 mg or 315 mg	60 min pre-test	Acute GPC supplementation significantly increased cognitive performance.
[103]	Prospective case–control study	15 patients, mild traumatic brain injuries (>18 yo)	Oral GPC 800 mg/d	8 wk	GPC improves cognitive function in patients with mild traumatic brain injuries.
[104]	RCT	36 individuals, T2DM and MCI (>60 yo)	Oral GPC 1200 mg/d	6 mo, 12 mo	GPC leads to slight improvement in cognitive function in T2DM patients with MCI at 6 mo and become significant at 12 mo.

Abbreviations: AD, Alzheimer’s disease; CDP, cytosine diphosphocholine; FASD, fetal alcohol spectrum disorders; GPC, L-*α*-glycerylphosphorylcholine; i.m., intramuscular(ly); i.v., intravenous(ly); MCI, mild cognitive impairment; PD, Parkinson’s disease; RCT, randomized controlled trial; SSRI, selective serotonin reuptake inhibitor; TIA, transient ischemic attack; and VaD, vascular dementia.

## Data Availability

No new data were created or analyzed in this study. Data sharing is not applicable to this article.

## Abbreviations

The following abbreviations are used in this manuscript:

Aβ	Amyloid-β-protein
AD	Alzheimer’s Disease
AI	Adequate intake
CETP	Cholesteryl-ester transfer protein
CVD	Cardiovascular disease
GPC	Glycerophosphocholine
GDE/Gpcpd	Glycerophosphodiesterase
FMO	Flavin-containing monooxygenases
HDL	High-density lipoprotein
LDL	Low-density lipoprotein
LPC	Lysophosphocholine
lyso-PLA1	Lysophospholipase A1
MDCK	Madin–Darby canine kidney
MRS	Magnetic resonance spectroscopy
NMR	Nuclear magnetic resonance
NTD	Neural tube defect
PC	Phosphatidylcholine
PLA2	Phospholipase A2
ROS	Reactive oxygen species
SAMP8	Senescence-accelerated mouse prone 8
SCFA	Short-chain fatty acids
TMA	Trimethylamine
TMAO	Trimethylamine N-oxide
T2DM	Type 2 diabetes mellitus
VLDL	Very-low-density lipoprotein
